# Frontal mucocele with an accompanying orbital abscess mimicking a fronto-orbital mucocele: case report

**DOI:** 10.1186/1472-6815-6-6

**Published:** 2006-04-18

**Authors:** Erdinc Aydin, Guzin Akkuzu, Babur Akkuzu, Banu Bilezikci

**Affiliations:** 1 Baskent University Faculty of Medicine, Department of Otorhinolaryngology, Ankara, Turkey; 2 Baskent University Faculty of Medicine, Department of Pathology, Ankara, Turkey; 3 Baskent University Faculty of Medicine, Department of Otorhinolaryngology & Head and Neck Surgery, 6. Cadde No:72/2, 06490 Bahcelievler-Ankara, Turkey

## Abstract

**Background:**

Mucoceles are slowly expanding cystic lesions with respiratory epithelium containing mucus most commonly affecting the frontal and ethmoidal sinuses. They are caused by obstruction of sinus ostium. Mucoceles exert pressure on the bony boundaries and due to the proximity to the brain and orbit extension to these areas are common.

**Case presentation:**

A case of a frontal mucocele with an accompanying orbital abscess mimicking a fronto-orbital mucocele is reported. A 77 year old female patient suffering from left sided proptosis and pain around the left eye was admitted to our department. She had a history of left frontal sinus mucocele one year ago that was offered an osteoplastic frontal sinus surgery that the patient refused. Patient had limitation of eye movements. Fundoscopic examination revealed a minimal papilledema. Coronal computerized tomography and orbital magnetic resonance imaging showed a frontal mucocele with suspicious erosion of the orbital roof and a superiorly localized extraconal mass displacing the orbit lateroinferiorly. Frontal and orbital masses had similar intensities. Thus surgery was planned for a fronto-orbital mucocele. During surgery no defect was found on the orbital roof. Frontal mucocele and orbital cystic mass was removed separately. Pathological examination showed a frontal mucocele and an orbital abscess wall. Postoperatively eye movements returned to normal and papilledema resolved.

**Conclusion:**

Fronto-orbital mucoceles are commonly encountered pathologies, but frontal mucocele with an orbital abscess is a rarely seen and should be kept in mind because their treatments differ.

## Background

Paranasal mucoceles are slowly expanding cystic lesions with pseudostratified columnar epithelium in the setting of a background of chronic inflammation filled with inspissiated mucus exerting pressure on the normal boundaries of the sinus due to the obstruction of sinus ostium [1]. The mucoceles are usually filled with clear to yellowish thick mucoid secretions [2]. The obstruction can be caused by congenital anomalies, allergy, infection, trauma, surgical intervention in the nose and neoplasms [3]. The pressure exerted by the mucocele can cause expansion of the sinus, thinning of the bony wall, and finally extension through the weakest point to the adjacent important structures namely orbit and cranial cavity [2]. Significant morbidity and potential mortality may ensue if mucoceles are allowed to grow. Such advanced mucoceles present challenge in their surgical management.

## Case report

**Figure 1:**
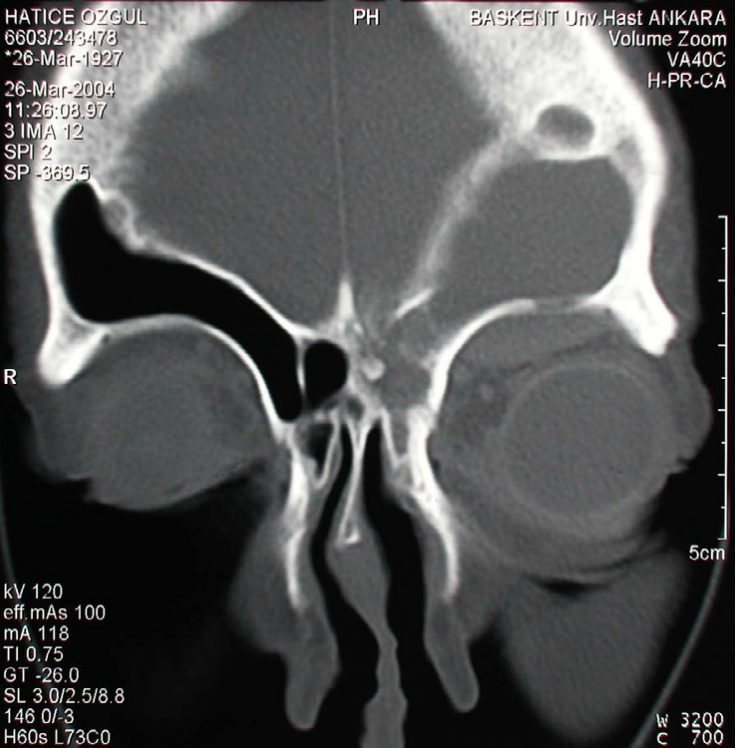
Coronal paranasal computerized tomography demonstrating a frontal mucocele and an intra orbital cystic mass having similar intensities and a suspected area of communication through the bony roof of the orbit.

**Figure 2:**
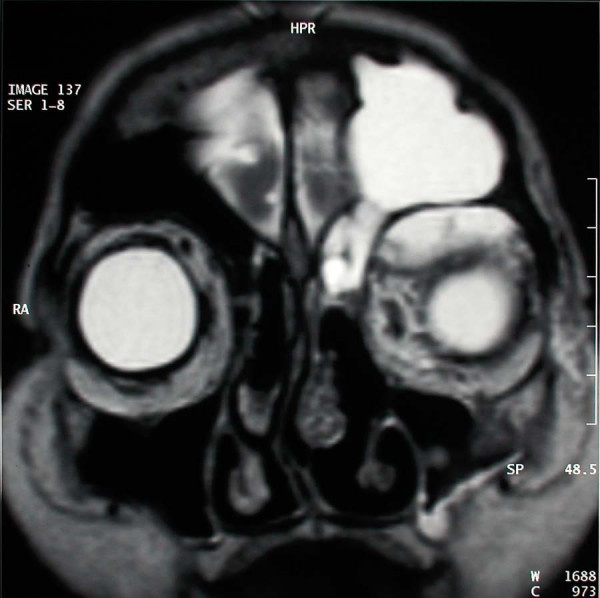
Coronal magnetic resonance imaging demonstrating a frontal mucocele and an orbital cystic mass.

**Figure 3:**
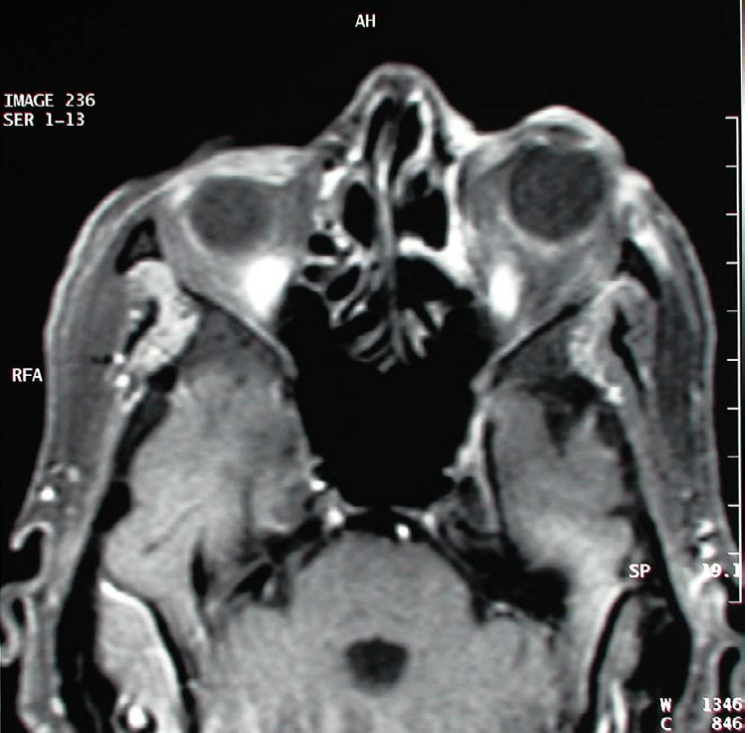
Axial magnetic resonance imaging demonstrating avert proptosis and lateral displacement of the orbit.

**Figure 4:**
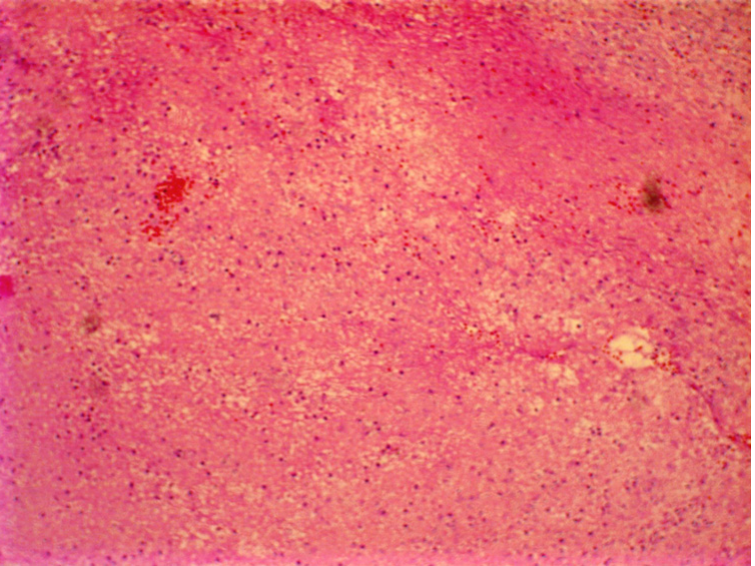
Microscopically, accumulation of inflammatory exudate, mucin secretion and the wall of mucocele composed of granulation tissue were seen.

**Figure 5:**
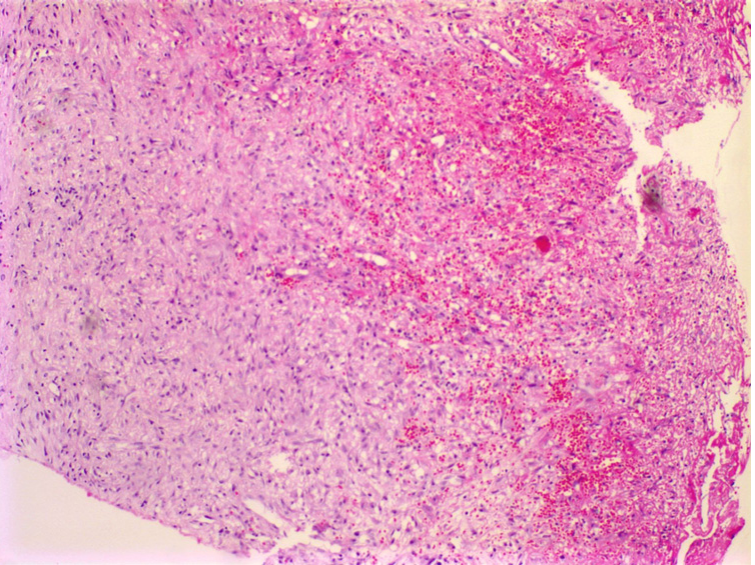
Microscopicaly orbital abses wall is seen composed of fibrosis and inflamatory cells lacking a mucosal lining.

**Figure 6:**
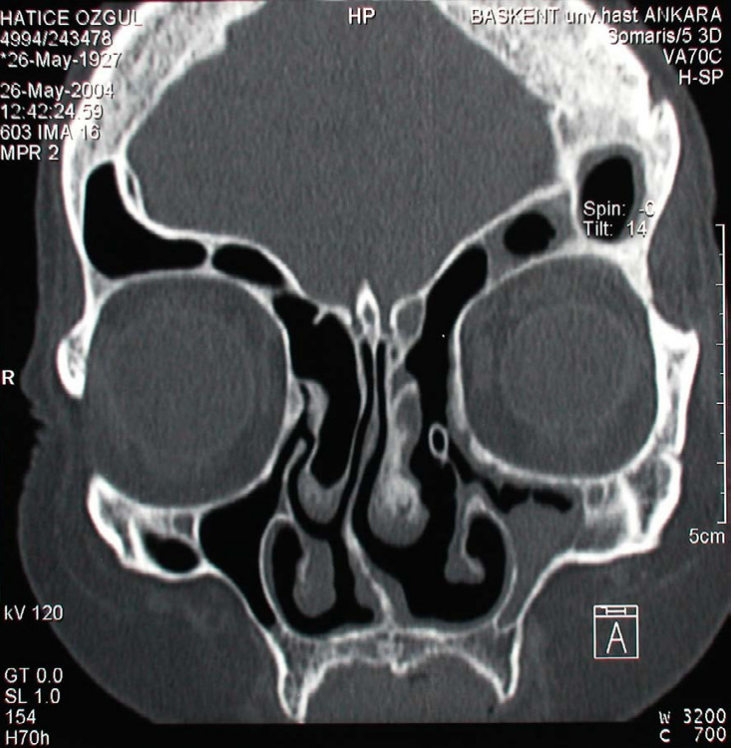
Postoperative coronal paranasal tomography demonstrating an aerating frontal sinus with a patent frontal recess that has a silicone stent in place.

A 77 year old female patient suffering from left sided proptosis and pain around the left eye was admitted to our department. She had a history of left frontal sinus mucocele one year ago that was offered an osteoplastic frontal sinus surgery that the patient refused. Patient had limitation of eye movements. Fundoscopic examination revealed a minimal papilledema. Coronal computerized tomography (Figure 1) and orbital magnetic resonance imaging (Figure 2, 3) showed a frontal mucocele with suspicious erosion of the orbital roof and a superiorly localized extraconal mass displacing the orbit lateroinferiorly. Frontal and orbital masses had similar intensities. Thus surgery was planned for a fronto-orbital mucocele. During surgery no defect was found on the orbital roof. Frontal mucocele and orbital cystic mass was removed separately. Pathological examination showed a frontal mucocele (Figure 4) and an orbital abscess wall (Figure 5). Postoperatively eye movements returned to normal and papilledema resolved. On early postoperative paranasal tomography frontal recess was patent with an aerating frontal sinus and inflammation of the eye was resolving (Figure 6).

## Discussion

Sites of occurrence of mucoceles are the maxillary sinus, frontal sinus, anterior ethmoidal sinus and rarely the posterior ethmoidal sinus and sphenoidal sinus [4]. The pathophysiology of frontoethmoid mucoceles has been lightened in experimental studies and by clinical observations that the trapped mucosa in the frontal and ethmoidal sinuses after obstruction of sinus ostia [5]. They may occur at any age, but most of them are seen between the fourth and seventh decades. They are seen similarly at both sexes. Frontoethmoid mucoceles cause outward and downward displacement of the globe and are often associated with a palpable mass in the superonasal and medial canthal region. The expanding mass lesion may cause proptosis, restriction of eye movements, diplopia, visual loss, retroorbital pain or headache.

The mucoceles of the frontal sinus may disrupt the medial canthal ligament and the orbital roof in which surgical interventions should include the reconstruction of these anatomic structures [6,7]. Many surgical approaches to frontal mucoceles have been defined but we preferred a combined endoscopic and external approach for the treatment of frontal mucocele and the orbital abscess suspected to be a fronto orbital mucocele. If we new that orbital mass was an abscess instead of a mucocele before the operation our approach would have been different. Instead of entering the orbit and removing the mass a long term course of antibiotics with drainage would have been preferred.

## Conclusion

Fronto-orbital mucoceles are commonly encountered pathologies, but frontal mucocele with an orbital abscess is a rarely seen and should be kept in mind because their treatments differ.

## Competing interests

The author(s) declare that they have no competing interests.

## Authors' contributions

EA carried out the medical care and surgery of the patient, participated in the design, writing and drafting of the article.

BA carried out the medical care and surgery of the patient, participated in the design, writing and drafting of the article.

GA participated in the design, writing and drafting of the article.

BB carried out the histopathological investigation and participated in the design, writing and drafting of the article.

All authors read and approved the final manuscript.

## Pre-publication history

The pre-publication history for this paper can be accessed here:


